# Identification and Expression Analysis of the CLE Gene Family in Sesame

**DOI:** 10.3390/ijms27198536

**Published:** 2026-09-24

**Authors:** Hailan Su, Chunji Xie, Bin Feng, Guoxian Sun, Jie Luo, Lintao Wu

**Affiliations:** 1 School of Biological Sciences, Guizhou Education University, Guiyang 550018, China; 2522060003@gznc.edu.cn (H.S.); sgx571263@163.com (G.S.); 2 Oil Research Institute, Guizhou Academy of Agricultural Sciences, Guiyang 550006, Chinafengbbing@126.com (B.F.); 3 School of Biology and Agricultural Science and Technology, Zunyi Normal University, Zunyi 563006, China; luo240410@163.com

**Keywords:** *Sesamum indicum*, *CLE* gene family, water stress

## Abstract

The *CLE* (CLAVATA3/EMBRYO SURROUNDING REGION-related) small peptides constitute a large supergene family in plants and are extensively involved in plant stress responses and the regulation of growth and development. Light, water, gravity, and mechanical trauma play critical roles in maintaining normal plant growth and development; however, there have been no reports as to whether *CLE* genes are involved in the responses of sesame to these forms of stress. In this study, the *CLE* gene family in Zhongzhi No. 13 was systematically identified. Stress induction was performed using light and gravity, and the expression patterns in various tissues and organs were analyzed by means of transcriptome sequencing. Concurrently, the water stress response published in the NCBI database was queried and analyzed. The results showed that a total of 12 *SiCLE* genes exist in the genome of Zhongzhi No. 13, distributed on six chromosomes. Each gene encodes a signal peptide at its N-terminus and a conserved motif at its C-terminus. The majority of *SiCLE* genes exhibit a single-exon structure. The *SiCLE* genes encode proteins ranging from 72 to 133 amino acids in length. Most of these proteins are basic, hydrophobic, and unstable. An analysis of promoter cis-acting elements revealed that the majority of *SiCLE* gene family members harbor response elements associated with light, phytohormones, stress, and growth and development. Transcriptome analysis revealed that *SiCLE4*, *SiCLE5*, *SiCLE8*, and *SiCLE10* exhibit differential expression under water stress, light stress, and gravity stress/mechanical damage conditions. These results provide a solid foundation for future investigations aimed at elucidating the biological roles of the *CLE* gene family in both abiotic stress responses and the regulation of growth and development.

## 1. Introduction

Sesame (*Sesamum indicum* L.) is an important crop in China, possessing high nutritional and economic value, and is a major source of high-quality oil and edible seeds [1]. The oil content of sesame seeds ranges from 50% to 60%, and they are also abundant in several natural antioxidants, including sesamol and vitamin E [2]. However, sesame is susceptible to abiotic stresses such as drought and water stress during production, which seriously affect its yield. Water is essential for plant growth; plants perceive soil water conditions via their root systems and respond to drought or water stress through various mechanisms, including stomatal movement and osmotic adjustment [3]. Light not only provides the energy required for plant photosynthesis but also functions as a crucial signal regulating plant morphogenesis and physiological metabolism [4]. In response to stress, the plant shade avoidance response can regulate plant growth, leading to earlier flowering, a shorter vegetative growth period, and faster plant growth [5,6]. Gravity is the basis for the tropistic movements of plant roots and shoots: starch granules within plant cells act as gravity sensors to perceive gravity’s direction, and through asymmetric auxin distribution via polar transport, they induce roots to grow toward gravity (positive gravitropism) and shoots to grow away from gravity (negative gravitropism) [7]. Plant *CLE* genes can participate in various stress response processes, and their response mechanisms differ under different stress conditions [8]. Therefore, this study systematically identified and characterized the *CLE* gene family in sesame, aiming to further elucidate the response mechanisms of its members under water, gravity, and light stress and mechanical trauma conditions.

Plant peptide signaling molecules, which are small-molecule polypeptides synthesized endogenously by plants, possess specific functional amino acid compositions and exhibit simple structural characteristics. They are important plant growth regulators and play a key role in intercellular signal transduction in plants [9,10]. The *CLE* gene family encodes plant-specific peptide hormones and represents one of the largest known peptide families in plants [11,12]. Previous research has demonstrated that *CLE* proteins generally consist of a relatively conserved N-terminal signal peptide and a variable C-terminal mature peptide region. Furthermore, a highly conserved *CLE* domain, typically 12–13 amino acids in length, is located at the C-terminus [12,13]. *CLE* small peptides play a crucial role in precisely regulating plant growth and development, for example, in the regulation of root apical stem cell maintenance and root development [14]. *CLE* gene family members have been extensively identified and characterized in a wide range of plant species, such as *Oryza sativa* (rice) [15], *Zea mays* (maize) [16], and *Arabidopsis thaliana* [17]. For instance, under drought stress, root-synthesized *AtCLE25* serves as a long-distance signal. It is perceived by BAM (Barely Any Meristem) receptors in leaves to induce NCED3-dependent ABA biosynthesis and stomatal closure, reducing transpiration water loss and consequently improving the water status of root tissues at the whole-plant level [17,18]. Additionally, *AtCLV3* influences the shoot apical meristem (SAM) stem cell number via a negative feedback loop involving the WUSCHEL gene [12]. In rice, FCP2 (also known as *OsCLE50*) modulates root apical meristem (RAM) activity and late metaxylem formation [15]. The expression level of *ZmCLE7* (the ortholog of *Arabidopsis* CLV3) in the maize meristem is directly related to the number of kernel rows per maize ear [16]. In soybean (*Glycine max*), the majority of *GmCLE* genes are involved in regulating both salt and drought stress responses, and they also play roles in root nodule formation and development [19]. In summary, *CLE* genes play key regulatory functions in plant growth, development, and abiotic stress responses, providing important genetic resources for crop molecular breeding.

Nevertheless, no studies have yet been reported on the roles of *CLE* genes in the growth, development, and stress adaptation of sesame. Consequently, the number, structural features, phylogenetic relationships, and expression patterns of these family members remain unclear. The *CLE* peptide family represents a well-characterized group of plant short-range signaling peptides, controlling stem cell fate and participating in diverse biological processes, including plant growth, development, and environmental responses [20]. This study systematically identified members of the sesame *CLE* gene family and analyzed their sequence characteristics, evolutionary relationships, and expression patterns, thereby providing a theoretical basis for exploring the molecular regulatory mechanisms of sesame growth and development.

## 2. Results

### 2.1. Genome-Wide Identification of SiCLE Gene Family Members and Physicochemical Characterization in Sesame

We used the protein sequences of *Arabidopsis thaliana AtCLE* genes as queries. However, since *CLE* proteins are short peptide precursors with relatively short conserved functional motifs, to minimize the presence of potential false-negative members, we first performed BLAST searches, which yielded 19 putative *SiCLE* gene family members. These 19 candidates were then subjected to domain prediction, and two genes lacking the *CLE* domain were excluded, resulting in 17 members. Next, signal peptide prediction analysis was carried out on these 17 members, and five genes lacking an N-terminal signal peptide were removed, leaving 12 *SiCLE* gene family members. Finally, conserved motif analysis was conducted on these 12 candidates. All of them were found to contain a conserved motif at the C-terminus, confirming them as bona fide *SiCLE* members. Therefore, a total of 12 *SiCLE* gene family members were identified in sesame. According to their order along the chromosomes, they were renamed *SiCLE1* to *SiCLE12*, with the prefix “Si” representing the sesame species (*S. indicum*) (Table 1).

According to the protein physicochemical property analysis, the *SiCLE* genes encode proteins ranging in length from 72 to 133 amino acids. Their isoelectric points range from 6.9 to 11.2; except for the *SiCLE3* protein, which has an isoelectric point below 7 and is classified as an acidic protein, they are all basic proteins. The relative molecular masses range from 8.29066 to 15.00339 kDa, with *SiCLE11* being the smallest and *SiCLE2* the largest. The instability index of the *SiCLE* proteins ranges from 36.27 to 86.27. Apart from *SiCLE11* and *SiCLE12*, which have instability indices below 40, all family members exhibit indices greater than 40. An analysis of protein solubility fractions indicated that only *SiCLE1* and *SiCLE3* exhibit a GRAVY (grand average of hydropathicity) value greater than 0 and are thus classified as hydrophilic proteins. In contrast, all other *SiCLE* family members are hydrophobic proteins (Table 1). Subcellular localization analysis revealed that sesame *SiCLE* proteins are mainly localized in the cytoplasm, chloroplasts, and vacuoles, while a small fraction of the expressed proteins are secreted to the extracellular matrix.

### 2.2. Chromosomal Distribution and Phylogenetic Analysis of SiCLE Genes in Sesame

Chromosomal localization showed that the 12 *SiCLE* genes are unevenly distributed across six chromosomes (Figure 1). Specifically, chromosome 8 contains the highest number, with four *SiCLE* genes. Chromosome 2 carries three genes, and chromosome 6 carries two. Chromosomes 12, 15, and 16 each harbor a single gene. Conversely, chromosomes 1, 3, 4, 5, 7, 9, 10, 11, 13, and 14 (nine chromosomes in total) have no *SiCLE* genes (Figure 1).

To further elucidate the evolutionary relationships within the sesame *CLE* gene family, a phylogenetic tree was constructed using the neighbor-joining (NJ) method. This analysis incorporated the amino acid sequences of 12 *SiCLE* proteins, 32 *AtCLE* proteins from *Arabidopsis thaliana*, 39 *GmCLE* proteins from soybean (*Glycine max*), and nine *AhCLE* proteins from peanut (*Arachis hypogaea*) (Figure 2). The 92 *CLE* proteins were divided into six groups. Group I contained 17 *AtCLE*, six *SiCLE*, and three *AhCLE* members. Group II included four *SiCLE*, five *AtCLE*, one *GmCLE*, and three *AhCLE* members. Group III contained 16 *AtCLE*, two *SiCLE*, and two *AhCLE* members. Group IV contained only one *AtCLE* member. Groups V and VI contained only soybean *GmCLE* members. Based on the above analysis, the *CLE* gene families of sesame, *Arabidopsis*, and peanut show stronger evolutionary conservation, while the *CLE* gene families of sesame and soybean may have undergone divergence during evolution, with soybean potentially exhibiting significant species-specific differentiation.

### 2.3. Analysis of Gene Structure and Conserved Motifs of SiCLE Genes in Sesame

According to the visualization results for the *SiCLE* gene structures (Figure 3), the gene sequence lengths within the *SiCLE* family exhibit relatively minor overall variation, with most sequences ranging from 200 to 400 bp. Notably, *SiCLE4* possesses the longest DNA sequence, exceeding 1 kb. The majority of *SiCLE* genes possess a single-exon structure, with only *SiCLE4* and *SiCLE2* each containing one intron. Furthermore, all identified *SiCLE* genes are devoid of both 5′ and 3′ untranslated regions (UTRs).

To further characterize the structural features of sesame *CLE* genes, three conserved motifs were identified using the MEME online tool (Figure 3). All genes of the *SiCLE* family possess motif 1 at their C-terminus, indicating that motif 1 is likely the most conserved gene sequence in the sesame *CLE* family, namely the *CLE* motif (SPKRVVPGGPDPIHH). The *SiCLE6*, *SiCLE9*, *SiCLE5*, *SiCLE7*, *SiCLE10*, and *SiCLE11* genes have the highest number of conserved motifs, each containing two. Notably, motif 3 (MDHYIQ) is exclusively found in *SiCLE5*, *SiCLE7*, *SiCLE10*, and *SiCLE11*. As shown in Figure 3A, *SiCLE* genes located on the same small branch of the phylogenetic tree share similar conserved motifs. For example, both *SiCLE6* and *SiCLE9* possess the specific motif 2 (PPPCRDGDDDIDPRYG). This observation suggests that *SiCLE* genes belonging to the same clade may share analogous biological functions.

### 2.4. Analysis of Cis-Acting Elements in the Sesame SiCLE Gene Promoters

An analysis of cis-acting elements in the promoter regions of *SiCLE* genes revealed that members of the *SiCLE* gene family possess a large number of cis-acting elements, mainly including light-responsive elements, phytohormone-responsive elements, stress-responsive elements, and elements related to growth and development regulation [21]. The identified phytohormone-responsive elements include those responsive to salicylic acid (SA), auxin, abscisic acid (ABA), and gibberellin (GA) (Table 2). Specifically, *SiCLE1* possesses only a single abscisic acid (ABA)-responsive element, whereas *SiCLE10* contains the largest number of such elements (14) (Figure 4). These observations indicate that the expression of the *SiCLE* gene family is likely interconnected with the endogenous levels of diverse plant hormones. The stress-responsive elements identified in the promoters include those involved in low-temperature response, anaerobic induction, and hypoxia-specific induction, as well as defense and stress responses (Table 2). *SiCLE12* possesses only a single defense and stress response element, whereas *SiCLE7* and *SiCLE11* contain the largest number of stress-responsive elements (eight) (Figure 4). The *SiCLE* gene family is hypothesized to be involved in multiple stress tolerance-related physiological processes in sesame. Growth and development-associated regulatory elements include those involved in meristem development and circadian rhythm control (Table 2). Except for *SiCLE7* and *SiCLE8*, which are devoid of such elements, all *SiCLE* family members harbor growth- and development-related cis-elements (Figure 4). It is speculated that *SiCLE* gene family members may be involved in meristem development and play important roles in the regulation of circadian rhythms during sesame growth. The promoter regions of all *SiCLE* gene family members are equipped with light-responsive elements. *SiCLE1* possesses the greatest number of light-responsive elements (17) and also the highest count of Box 4 elements. In contrast, *SiCLE11* and *SiCLE12* contain the fewest light-responsive elements (six) (Figure 4). These prediction results suggest that the expression of this gene family may be generally subject to potential light regulation.

### 2.5. Collinearity Analysis of SiCLE Genes 

To gain a deeper understanding of the sesame *CLE* gene family’s evolutionary relationships, collinearity analysis was performed within the sesame genome itself (Figure 5) and between the sesame genome and those of *Arabidopsis* and *Perilla* (Figure 6) using TBtools software (v2.363). Through intraspecific collinearity analysis of the sesame *CLE* gene family, two pairs of genes exhibiting segmental duplication were identified among the 12 *SiCLE* members: *SiCLE4* and *SiCLE10*, and *SiCLE1* and *SiCLE12*. Interestingly, *SiCLE4* and *SiCLE10* belong to the same subgroup, while *SiCLE1* and *SiCLE12* belong to another subgroup. This suggests that these chromosomal segments may have undergone duplication during evolution but have not yet fully diverged, and their functions may exhibit redundancy (Figure 5). This indicates that segmental chromosomal duplications occur during plant genome evolution, leading to gene duplication. The interspecific collinearity results showed that there is one pair of orthologous genes between *AtCLEs* and *SiCLEs* and six pairs between *AtCLEs* and *PfCLEs*, indicating that sesame shares an evolutionary homologous relationship with *Arabidopsis* and *Perilla* but that the divergence time between sesame and *Arabidopsis* is relatively distant (Figure 6).

### 2.6. Expression Patterns of the CLE Gene Family in Sesame

#### 2.6.1. Expression Analysis of Sesame *CLE* Genes in Response to Water Stress

The dataset GSE133186, which was downloaded from the NCBI GEO public database, is a sesame gene expression profile dataset generated under water stress. The expression dataset describes two sesame cultivars: ZZM2541 (R2G), characterized by strong water tolerance, and Ezhi-2 (EG), which is relatively sensitive to water stress. Expression analysis detected four members of the sesame *CLE* gene family (*SiCLE4*, *SiCLE5*, *SiCLE8*, and *SiCLE10*), whereas the remaining eight genes (*SiCLE1*, *SiCLE2*, *SiCLE3*, *SiCLE6*, *SiCLE7*, *SiCLE9*, *SiCLE11*, and *SiCLE12*) showed no detectable expression. According to a heatmap analysis of the sesame *CLE* gene expression profile data, following 4 and 48 h of water stress, *SiCLE5* expression was upregulated in the tissues of both sesame cultivars. This finding indicates that *SiCLE5* may participate in the regulation of water stress responses and likely plays a significant role in sesame’s response to water stress (Figure 7). After 4 or 8 h of water stress, the expression levels of *SiCLE4*, *SiCLE8*, and *SiCLE10* were downregulated in the tissues of both sesame cultivars. For example, after R2G and EG were subjected to water stress for 4 h, the expression levels of *SiCLE4* and *SiCLE10* decreased in both cultivars, indicating that 4 or 8 h of water stress may inhibit the expression of these genes, thereby affecting the physiological regulation of sesame growth and development (Figure 7).

#### 2.6.2. Expression Analysis of Sesame *CLE* Genes in Response to Light Stress, Gravity Stress, and Mechanical Trauma

To analyze the roles of *SiCLE* genes under different abiotic stresses, transcriptome data from sesame subjected to light and gravity stress were analyzed. Sesame seedlings were exposed to either high-light or low-light conditions. Samples were collected on the second and sixth days of light treatment to assess the expression changes of the *SiCLE* gene family members across three types of tissue: roots, stems, and leaves. As shown in Figure 8, most *SiCLE* gene family members showed certain expression levels in all three tissue types, and the expression changes for *SiCLE2*, *SiCLE4*, *SiCLE5*, and *SiCLE10* were relatively significant across the three types. Following two and six days of exposure to either high-light or low-light conditions, *SiCLE2* transcript levels increased in roots, stems, and leaves, with the sole exception of a decrease in leaves on day two of low-light treatment. *SiCLE8* transcript levels were reduced in roots, stems, and leaves. *SiCLE4* expression decreased in leaves but increased in roots. *SiCLE5* expression was elevated in both roots and leaves. *SiCLE10* expression was upregulated in stems and leaves. On day six of both light treatments, *SiCLE4* and *SiCLE5* showed increased expression in stems, while *SiCLE10* expression was elevated in roots. These observations suggest that, under the influence of light induction, *SiCLE4*, *SiCLE5*, *SiCLE8*, and *SiCLE10* may be involved in regulating light stress responses and contribute substantially to sesame growth and development. *SiCLE1*, *SiCLE11*, and *SiCLE12* were expressed only in roots, suggesting that these genes may specifically participate in light stress response regulation within sesame roots (Figure 8).

Under gravity stress treatments at different relative centrifugal forces of 173× *g* (1000 rpm; 173× *g* corresponds to 173-fold gravitational acceleration), 1560× *g* (3000 rpm), and 4332× *g* (5000 rpm), tissue-specific gene expression was observed. Specifically, *SiCLE5* and *SiCLE11* exhibited upregulated expression in sesame roots, stems, and stem tips. *SiCLE10* expression increased in stems and stem tips but showed initial upregulation followed by downregulation in roots. *SiCLE4* and *SiCLE9* displayed distinct expression patterns across the three tissues depending on the rotational speed. Furthermore, *SiCLE1*, *SiCLE8*, and *SiCLE12* were expressed exclusively in roots and stems. These results indicate that the aforementioned genes may play a role in modulating the response to both gravity stress and mechanical trauma (Figure 9). Under treatments applying different relative centrifugal forces, the expression level of *SiCLE2* was downregulated in sesame roots, stems, and stem tips, indicating that gravity stress or mechanical trauma may inhibit the expression of this gene, thereby affecting the physiological regulation of sesame growth and development (Figure 9). During gravity stress induction, no expression of *SiCLE3* or *SiCLE6* was detected in sesame roots, stems, or shoot tips.

To validate the transcriptome data, five *SiCLE* genes showing significant differential expression (*SiCLE2*, *SiCLE4*, *SiCLE5*, *SiCLE8*, and *SiCLE10*) were selected. Sesame roots, stems, and shoot apices were subjected to gravity stress/mechanical wounding treatments at relative centrifugal forces of 173× *g*, 1560× *g*, and 4332× *g*. In parallel, three differentially expressed genes (*SiCLE2*, *SiCLE4*, and *SiCLE10*) were chosen to examine their expression levels in sesame roots, stems, and leaves after 2 d and 6 d of high-light and low-light treatments using qRT-PCR (Figure 10). The results showed that the expression trends of these genes across different tissues and treatments were highly consistent with the RNA-seq data, thus confirming the accuracy and reliability of the transcriptome sequencing results.

## 3. Discussion

The *CLE* gene family is recognized as one of the largest known peptide families [11] and is involved in numerous physiological processes governing plant growth and development. To date, analyses of the structure and function of the *CLE* gene family have been conducted in most dicotyledonous and monocotyledonous plants. *CLE* polypeptides are a class of small-molecule peptide hormones encoded by *CLE* genes and are widely present in plants [22]. They can directly participate in plant responses to abiotic stress. For example, in *Arabidopsis*, the expression of *AtCLE1*, *AtCLE13*, *AtCLE14*, and *AtCLE17* is induced under nitrogen deficiency stress, and their expression suppresses lateral root growth [23]. Under drought stress conditions, *AtCLE25* functions as a long-distance signaling molecule transmitting root-originated dehydration signals to leaves to trigger drought responses [17]. In tomato, *SlCLE11* participates in arbuscular mycorrhizal (AM) symbiosis, acting as a negative regulator of mycorrhizal colonization [24]. Although significant progress has been made regarding the *CLE* gene family in model plants such as *Arabidopsis* and tomato, studies on *CLE* genes in the oil crop sesame have rarely been reported. In this study, by systematically identifying and characterizing the *CLE* gene family in sesame and analyzing its expression profiles under light, water, and gravity stress or mechanical trauma conditions, we aimed to further elucidate the functions of the *SiCLE* family members in response to environmental stress. These findings will provide a foundation for uncovering the stress tolerance roles of the *CLE* gene family in sesame.

In this study, the *CLE* gene family members in sesame were systematically identified. Through comparison with *Arabidopsis CLE* proteins, 19 *SiCLE* genes were initially identified. However, after further prediction of conserved motifs, signal peptides, and domains, only 12 *SiCLE* genes were found to possess both a signal peptide and a domain. Compared with the 32 *AtCLE* genes in *Arabidopsis* [8] and the 39 *GmCLE* genes in soybean [25], the number of genes identified in this study is relatively small, indicating that the number of *CLE* gene family members varies greatly among different species. The structural organization of the identified *SiCLE* proteins is similar to that observed in other plant species—a signal peptide is present at the N-terminus, a long variable region is located in the middle, and a plant-specific conserved *CLE* motif (SPKRVVPGGPDPIHH) resides at the C-terminus—indicating that the structure of *CLE* genes is conserved across different species [26]. Furthermore, previous research has demonstrated that the *CLE* motif is the minimal functional unit required for *CLE* peptides to activate downstream signaling and exert their regulatory effects [27,28]. Gene structure analysis revealed that among the sesame *CLE* gene members, only *SiCLE2* and *SiCLE4* contain introns, while all other genes are intronless, which is consistent with the relatively simple structural characteristic of *CLE* genes. Furthermore, the majority of *SiCLE* genes are single-exon genes, a finding consistent with previous studies in *Arabidopsis* [29].

According to a physicochemical property analysis of this gene family, the number of amino acids in its proteins ranges from 72 to 133, which is consistent with the characteristic short sequence length of small peptide hormones. *SiCLE3* has an isoelectric point below 7, but the isoelectric points of the other 11 *CLE* protein sequences are all above 7, indicating that sesame *CLE* genes are almost all basic in nature. The molecular weights of sesame *CLE* members range from 8.29 to 15.00 kDa, values that are comparable to those of *CLE* proteins characterized in other plant species. The instability index for *SiCLE* proteins ranges from 36.27 to 86.27. Only *SiCLE11* and *SiCLE12* have indices below 40, whereas all other members exhibit indices exceeding 40, suggesting that the majority of *SiCLE* proteins are unstable. According to a hydrophilicity analysis of *SiCLE* gene members, sesame *CLE* genes are almost all hydrophobic proteins. Subcellular localization analysis indicated that sesame *SiCLE* proteins are predominantly distributed in the extracellular matrix, chloroplasts, cytosol, and vacuoles, with only *SiCLE5* being localized in the cytosol. An analysis of the cis-acting elements in the promoters of *SiCLE* genes revealed that the promoter regions contain light-responsive elements, stress-responsive elements, plant growth- and development-responsive elements, and phytohormone-responsive elements such as those for gibberellin. This suggests that the *SiCLE* family may participate in abiotic stress regulation together with various plant hormones. Through analysis of water stress transcriptome data for sesame obtained from the GEO database, together with light and gravity stress transcriptome datasets generated by Novogene Bioinformatics Technology Co., Ltd. (Tianjin, China), the expression patterns of the *SiCLE* gene family members in different sesame tissues under various stresses were characterized at the transcriptome level. *CLE* genes were found to be expressed in almost all sesame tissues, including roots, stems, shoot tips, and leaves, albeit at relatively low levels; this may be related to the trace but highly efficient nature of small peptide hormones. The expression level of *SiCLE5* was significantly upregulated in sesame tissues under almost all water, light, gravity, and mechanical damage stress inductions. The *SiCLE8* gene showed significant expression in sesame tissues under water, light, and gravity stress or mechanical damage induction. *SiCLE2*, *SiCLE4*, and *SiCLE10* showed specific expression under light stress in sesame tissues, while *SiCLE11* showed specific expression under gravity stress. It is important to note that this study employed centrifugation to simulate gravity stress, a method that artificially increases gravitational acceleration and is widely used as a ground-based approach to investigate gravity-related signaling in plants [30]. Centrifugation-induced gravitational stress is not entirely equivalent to the classical gravitropic signaling pathway in plants. Gravitropism primarily senses the direction of the gravity vector through the sedimentation of statolith amyloplasts, leading to organ growth reorientation. In contrast, hypergravity generated via centrifugation can activate gravity-related responses but inevitably introduces mechanical stimulation, resulting in nonspecific mechanical stress effects [30,31]. Therefore, when interpreting the molecular data, it is critical to differentiate responses mediated specifically by the gravity signal from those elicited by mechanical or other stresses accompanying centrifugation. To fully disentangle these two types of responses, additional control experiments, including the use of gravity perception-deficient mutants and mechanoreceptor inhibitors, are required to further delineate the respective signaling pathways involved [30,31]. During the experiment, at a relative centrifugal force of 4332× *g*, some sesame seedlings exhibited wilting, the leaves showed slight chlorosis, and, in some plants, the root tips even became necrotic. These observations suggest that relative centrifugal forces exceeding 2000× *g* cause lethal mechanical loading and physical tissue damage in plants, rather than eliciting a physiological gravitational response. Accordingly, in our experimental setup, only the 173× *g* and 1560× *g* treatments were classified as gravity induction, while the 4332× *g* treatment was considered a combination of gravity stress and mechanical damage.

Phylogenetic analysis revealed that the *CLE* gene family members of sesame, *Arabidopsis*, peanut, and soybean are interlinked and distributed across different branches, indicating that *CLE* genes are highly conserved during evolution [12,32]. *SiCLE* and *AtCLE* genes residing within the same phylogenetic clade may share analogous biological functions. In *Arabidopsis*, *AtCLE45* and its specific receptor BAM3 affect the proliferation and differentiation of the protophloem, thereby influencing root meristem organization [23]. *SiCLE5* and *AtCLE45*, as well as *SiCLE2* and *AtCLE25*, are grouped within the same clade in the phylogenetic tree, suggesting evolutionary relatedness between them. Transcriptome data analysis revealed that *SiCLE4*, *SiCLE5*, *SiCLE8*, and *SiCLE10* exhibited differential expression under water stress, light stress, and gravity stress/mechanical damage conditions. *SiCLE2* and *SiCLE12* were differentially expressed under light stress, whereas *SiCLE1*, *SiCLE2*, *SiCLE11*, and *SiCLE12* exhibited distinct expression changes in response to gravity stress/mechanical wounding. Collectively, these findings provide a solid foundation for further elucidating the biological functions of the *CLE* gene family in abiotic stress responses and growth or developmental processes.

## 4. Materials and Methods

### 4.1. Experimental Materials

This study utilized the sesame cultivar ‘Zhongzhi 13’ as the experimental material. Following uniform seedling cultivation, healthy and uniformly growing seedlings were selected for use in subsequent experiments. The seedlings were divided into two treatment groups. One group of sesame seedlings was subjected to high-light and low-light induction, with light intensities of 40,000 lux and 15,000 lux, respectively. Both groups were maintained under a day/night temperature regime of 28 °C/16 °C, a photoperiod of 16 h light/8 h dark, and a relative humidity of 80%. The treatment lasted for 6 consecutive days, and samples were collected on the second and sixth days of light induction (one sample pooled from three seedlings, with three biological replicates). The second group received gravity stress treatment via centrifugation at 1000 rpm (gravity induction), 3000 rpm (gravity induction), or 5000 rpm (gravity combined with mechanical damage induction), generating centrifugal forces of approximately 173× *g*, 1560× *g*, and 4332× *g*, respectively (based on a rotor radius of 15.6 cm). The treatment lasted for 1 h, after which root, stem, and shoot apex tissues were harvested. However, it should be noted that under the 5000 r/min treatment condition, the plants showed some wilting, the leaves of the seedlings displayed slight chlorosis, and root tip necrosis was observed in some plants. All collected samples were immediately flash-frozen in liquid nitrogen and stored at −80 °C. Subsequently, they were sent to Novogene Bioinformatics Technology Co., Ltd. (Tianjin, China) for high-throughput sequencing.

### 4.2. Identification and Physicochemical Property Analysis of SiCLE Gene Family Members in Sesame

The genome data and GFF3 annotation file for ‘Zhongzhi 13’ were obtained from the Ensembl Plants database (https://plants.ensembl.org/index.html, accessed on 10 January 2026). The 32 previously reported *Arabidopsis CLE* genes were identified through a literature review and by consulting the TAIR database (https://www.arabidopsis.org, accessed on 10 January 2026). Relevant amino acid sequences were downloaded from NCBI (https://www.ncbi.nlm.nih.gov/, accessed on 10 January 2026). Local BLASTP searches were performed against the sesame genome protein database using TBtools-II software (v2.363) with the *AtCLE* protein sequences as queries, setting an E-value threshold of ≤1 × 10^−5^, to obtain candidate *CLE* gene sequences. The obtained sequences were submitted to the online tools SMART 9.0 (http://smart.embl-heidelberg.de/, accessed on 12 January 2026), SignalP 6.0 (https://services.healthtech.dtu.dk/services/SignalP-6.0/, accessed on 12 January 2026), and MEME Suite (https://meme-suite.org/, accessed on 12 January 2026) for domain, signal peptide, and conserved motif prediction. Sequences lacking a domain, conserved motif, or N-terminal signal peptide were removed, ultimately determining the *SiCLE* family members in sesame.

TBtools-II software (v2.363) was used to extract the structural information of each *SiCLE* gene from the sesame genome GFF3 annotation file and to calculate the physicochemical properties of the encoded *SiCLE* proteins, including their molecular weight, isoelectric point, amino acid number, and hydrophilicity. The online software WoLF PSORT (https://wolfpsort.hgc.jp/, accessed on 12 January 2026) was used to predict the subcellular localization of the proteins [33].

### 4.3. Chromosomal Localization and Phylogenetic Analysis of SiCLE Genes in Sesame

Chromosomal localization information for the *SiCLE* genes was extracted from the sesame genome annotation file using TBtools-II software, which was also used for subsequent visualization. The 12 identified *SiCLE* protein sequences, 32 *AtCLE* protein sequences, 9 peanut *AhCLE* protein sequences (https://pgr.itps.ncku.edu.tw/, accessed on 20 January 2026), and 39 soybean *GmCLE* protein sequences (https://www.ncbi.nlm.nih.gov/, accessed on 20 January 2026) were combined. Multiple sequence alignment was performed using Clustal W (v2.1) software. A phylogenetic tree was constructed using MEGA11.0 software with the neighbor-joining (NJ) method and a bootstrap value of 1000 replicates [34]. The tree was further refined using the online software Chiplot 2.0 (https://www.chiplot.online/unrootedTree.html, accessed on 20 January 2026).

### 4.4. Analysis of SiCLE Gene Structure and Conserved Motifs in Sesame

The annotation information for the *SiCLE* gene family was extracted from the sesame genome GFF3 annotation file, and the *CLE* protein sequences were submitted to MEME Suite (https://meme-suite.org/, accessed on 12 January 2026) for conserved motif prediction. The resulting XML file was saved, and TBtools-II software (v2.363) was used to visualize the gene structures and conserved motifs of the *SiCLE* genes.

### 4.5. Analysis of Cis-Acting Elements in the Promoters of Sesame SiCLE Genes

The 2000 bp sequence upstream of the start codon of each *SiCLE* gene was extracted as the promoter sequence using TBtools-II software (v2.363). These promoter sequences were then submitted to the Plant CARE online promoter analysis tool (http://bioinformatics.psb.ugent.be/webtools/plantcare/html/, accessed on 21 January 2026) [35] to detect cis-acting elements related to abiotic stress in the *SiCLE* genes. The results were visualized using TBtools-II software (v2.363).

### 4.6. Collinearity Analysis of SiCLE Genes in Sesame

The genome data files and GFF3 annotation files for sesame and *Arabidopsis* were obtained from the Ensembl Plants database (https://plants.ensembl.org/index.html, accessed on 10 January 2026), while the corresponding files for perilla were obtained from NCBI (https://www.ncbi.nlm.nih.gov/, accessed on 25 January 2026). Homologous gene pairs were identified using MCScanX (v1.3.1) within TBtools-II (v2.363), and comparative collinearity maps of sesame relative to *Arabidopsis* and *Perilla* were generated. Interspecific collinearity results were generated and visualized using the multiple collinearity plot tool in TBtools-II (v2.363). The McScanX software (v1.3.1) within TBtools-II (v2.363) was also used to investigate collinearity among sesame *CLE* genes and to draw intraspecific collinearity maps.

### 4.7. Expression Analysis of the CLE Gene Family in Sesame

The transcriptome expression profile data for sesame under light stress and gravity stress were provided by Novogene Bioinformatics Technology Co., Ltd. (Tianjin, China). The sesame expression profile dataset GSE133186 was downloaded from the NCBI GEO public database (https://www.ncbi.nlm.nih.gov/gds/, accessed on 21 January 2026) [36]. In the data analysis stage, raw reads were first subjected to quality control using Fastp software (v1.1.0) to filter out low-quality sequences and adapter contamination, thereby obtaining high-quality clean data [37]. The filtered reads were then mapped to the sesame reference genome using Hisat2 software (v2.2.1) for sequence alignment [38]. The featureCounts (v2.0.1) program was used to quantify the number of aligned reads per gene, and the quantification results from each sample were aggregated to generate an expression matrix [39]. Finally, the expression data were normalized using the Log2FPKM and Log2TPM methods. Based on gene IDs, the expression data for *CLE* gene family members were filtered from the expression profile datasets. Normalized FPKM and TPM values were averaged. Heatmaps were used to compare the expression differences for *CLE* family members under water, light, and gravity stresses. Gene expression heatmaps were generated using TBtools-II software (v2.363).

### 4.8. Quantitative Real-Time PCR Validation

From the transcriptome datasets, five *SiCLE* genes showing notable expression differences were identified. Gene-specific primers were subsequently designed using the NCBI Primer-BLAST tool (Table 3). Sesame Histone was selected as the reference gene [40]. Quantitative real-time PCR was carried out using Promega Go Taq qPCR Master Mix (Promega Corporation, Madison, WI, USA) according to the manufacturer’s instructions on a real-time PCR instrument. Each sample was run in three technical replicates, and the relative expression level of each target gene was calculated using the 2^−ΔΔCt^ method.

## 5. Conclusions

In this study, the *SiCLE* gene family in sesame was identified through bioinformatics analysis, and a total of 12 *SiCLE* genes were identified, distributed across six chromosomes. Analysis of the *SiCLE* protein sequences showed that their lengths range from 72 to 133 amino acids. The majority of *SiCLE* genes are intronless, exhibiting a single-exon structure, and all encode a conserved *CLE* motif and an N-terminal signal peptide. Transcriptome data analysis revealed that *SiCLE4*, *SiCLE5*, *SiCLE8*, and *SiCLE10* showed significant differential expression in sesame plants under water stress, light stress, and gravity stress/mechanical damage treatments. *SiCLE2* and *SiCLE12* exhibited clear expression differences under light stress, while *SiCLE1*, *SiCLE2*, *SiCLE11*, and *SiCLE12* were differentially expressed under gravity stress/mechanical damage. These results suggest that *SiCLE* genes may play important roles in the response of sesame to abiotic stresses such as light, water, and gravity.

## Figures and Tables

**Figure 1:**
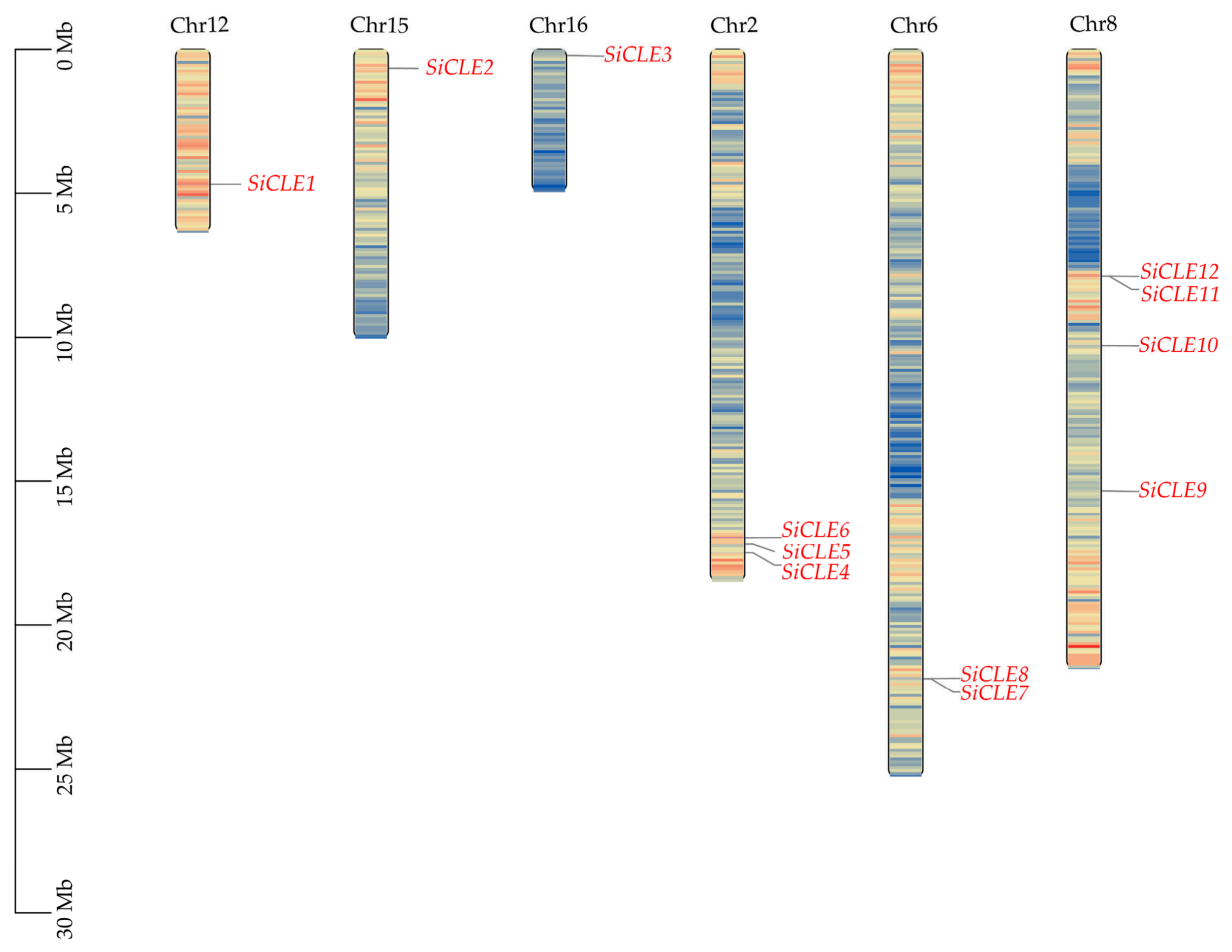
Chromosomal location analysis of *SiCLE* genes. Note: The colors on the chromosomes indicate the gene density of all genes. A redder color indicates higher gene density, while a bluer color indicates lower gene density.

**Figure 2:**
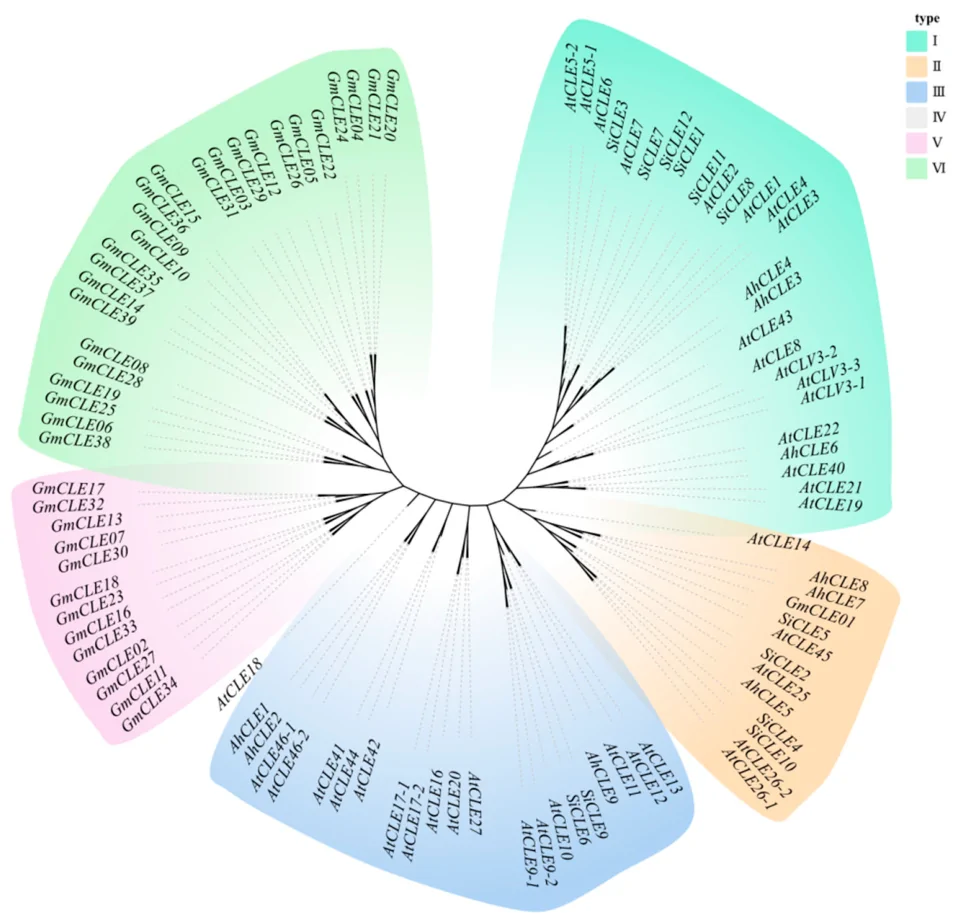
Phylogenetic analysis of Sesame, *Arabidopsis thaliana*, Peanut, and Soybean *CLE* genes. This unrooted phylogenetic tree was constructed by MEGA 11.0 using the neighbor-joining method with 1000 bootstrap replicates. All *CLE* genes were clustered into six subgroups (I–VI. Subgroup I mainly contains *CLV3* homologs responsible for shoot apical meristem maintenance. Subgroup II genes play a role in root development and drought stress responses (represented by *AtCLE25*). SubgroupIII corresponds to TDIF-type *CLEs* that regulate vascular cambium proliferation (represented by *AtCLE41/44*). Subgroup IV is related to embryo and seed development. Subgroups V and VI are dominated by soybean *CLE* genes, which have undergone species-specific expansion in soybean and are involved in nodulation regulation and plant growth.

**Figure 3:**
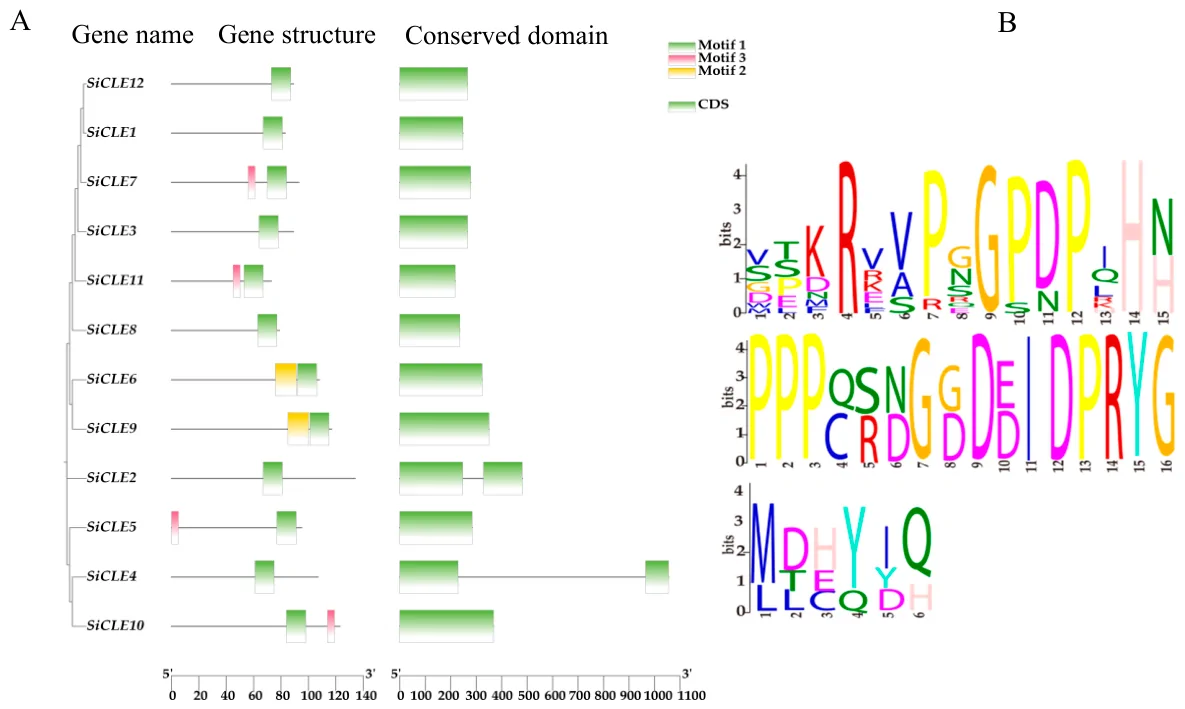
Conserved motif distribution and gene structure of *SiCLE* genes. (**A**) A schematic diagram of gene structure analysis and protein domain analysis results for the *SiCLE* gene family members; (**B**) A schematic diagram of the conserved *CLE* motifs of the *SiCLE* gene family.

**Figure 4:**
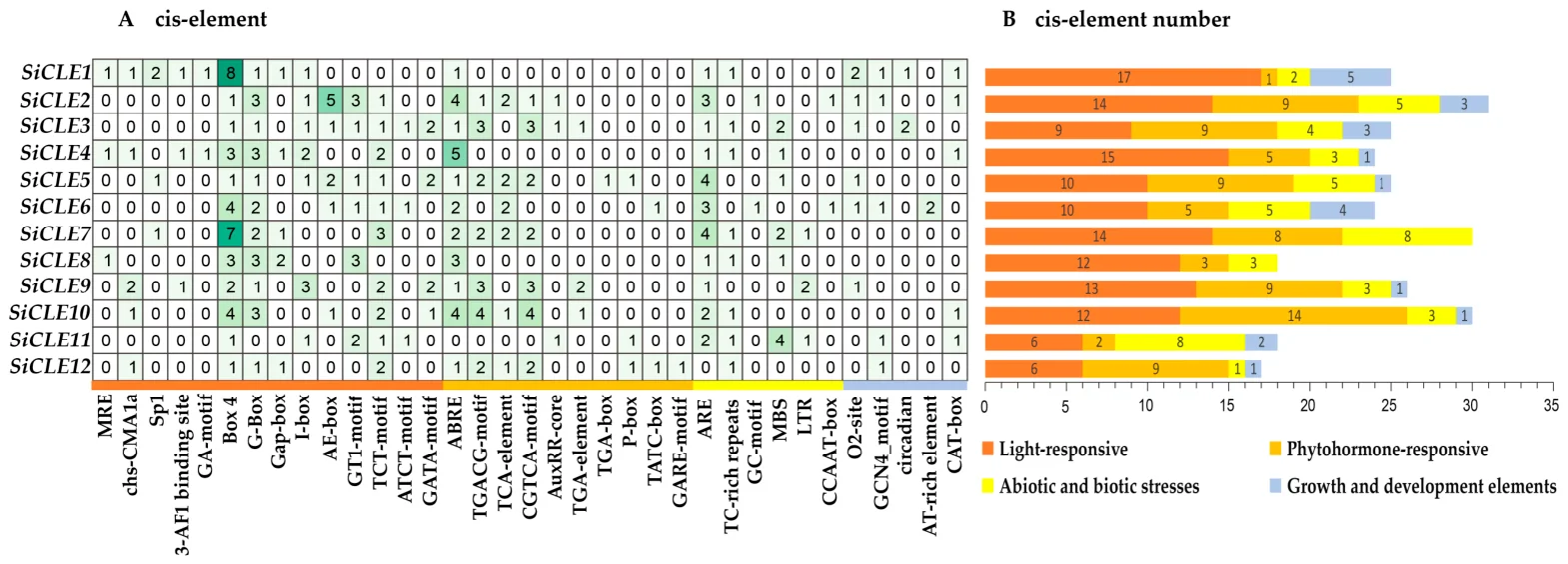
Cis-acting element distribution of the *SiCLE* gene family.

**Figure 5:**
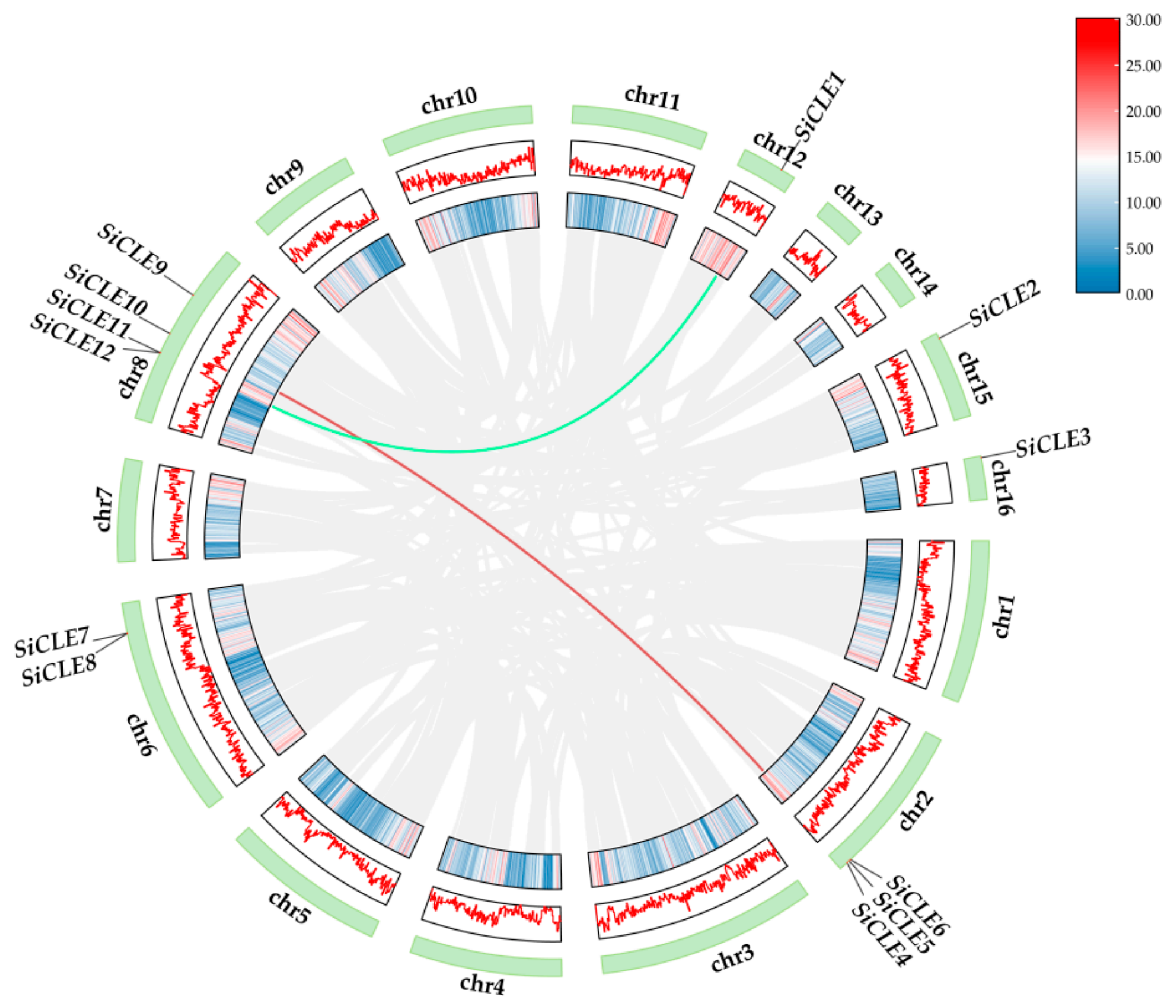
The intraspecies collinearity of the *SiCLE* gene family.

**Figure 6:**
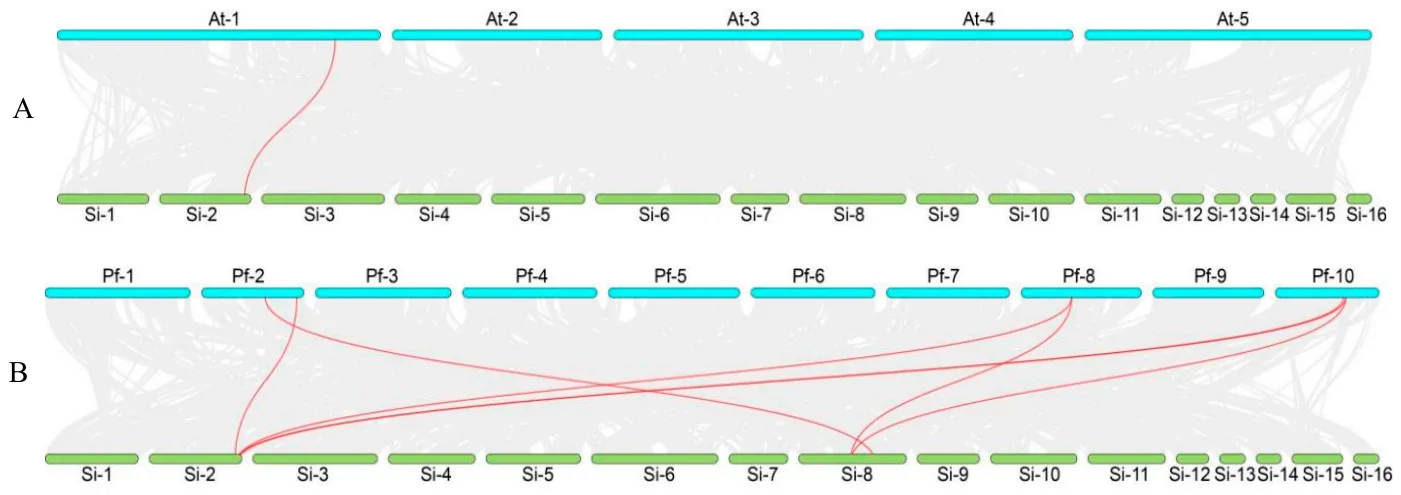
(**A**) Collinearity analysis of *CLE* genes of Sesame and *Arabidopsis*. (**B**) Collinearity analysis of *CLE* genes of Sesame and *Perilla*.

**Figure 7:**
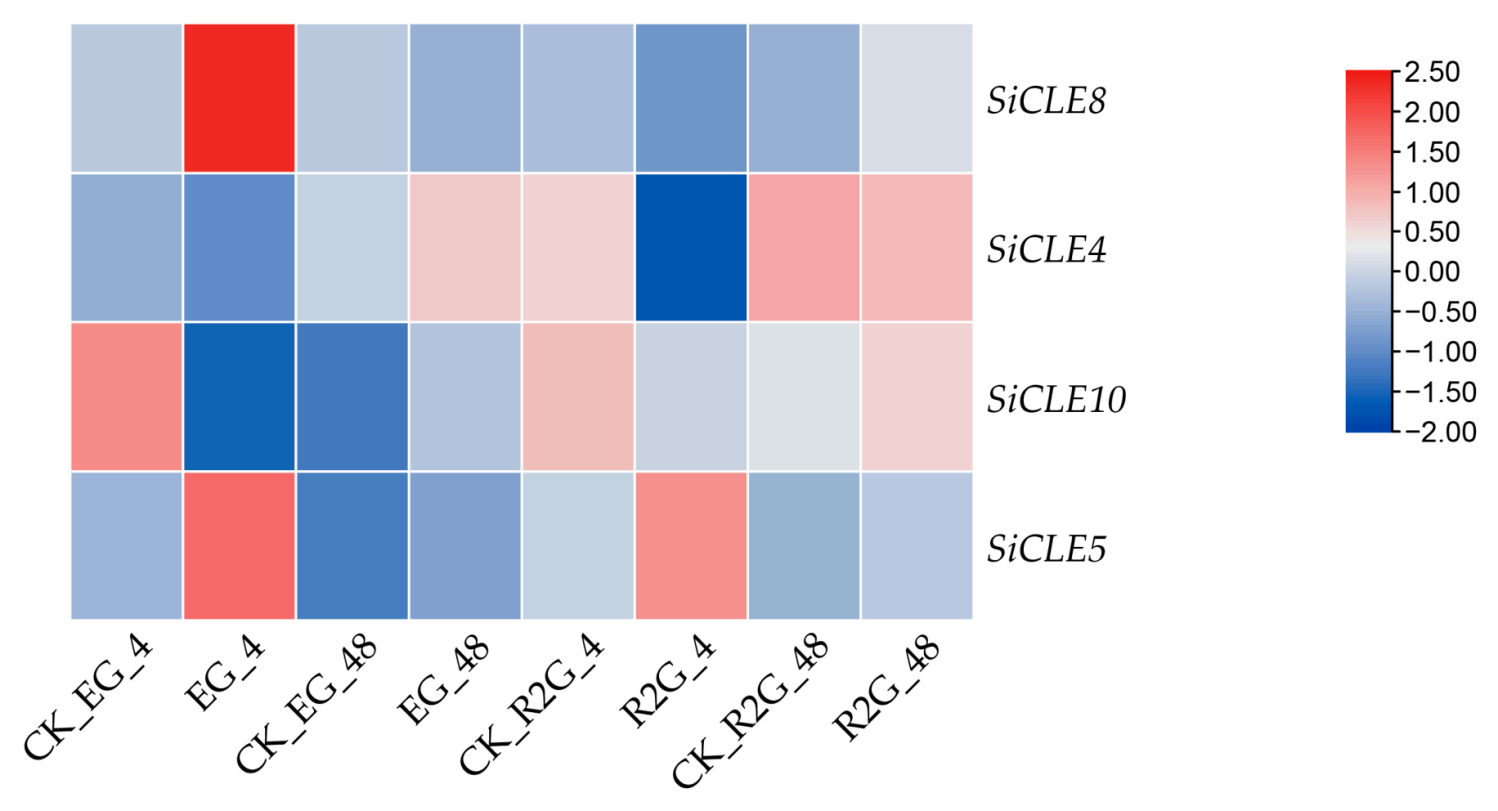
Expression analysis of *CLE* gene family members in response to water stress in sesame (plant materials derived from ZZM2541 and Ezhi-2).

**Figure 8:**
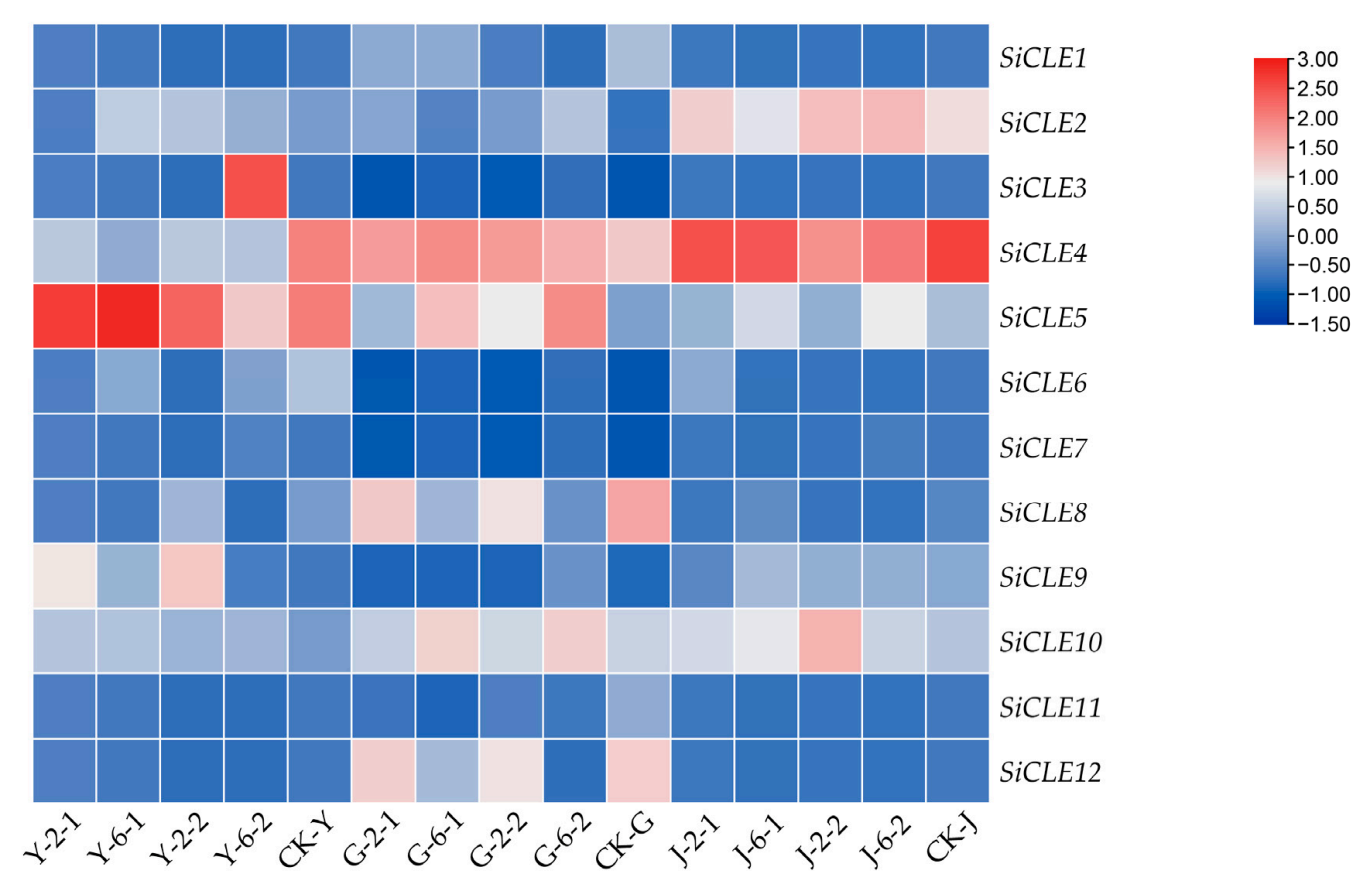
Expression analysis of *CLE* gene family members in response to light stress in sesame. G-2/6-1, J-2/6-1, and Y-2/6-1 denote the expression variations in roots, stems, and leaves at days 2 and 6 following high-light treatment. G-2/6-2, J-2/6-2, and Y-2/6-2 denote the expression variations in roots, stems, and leaves at days 2 and 6 following low-light treatment. CK-G/J/Y are the control samples.

**Figure 9:**
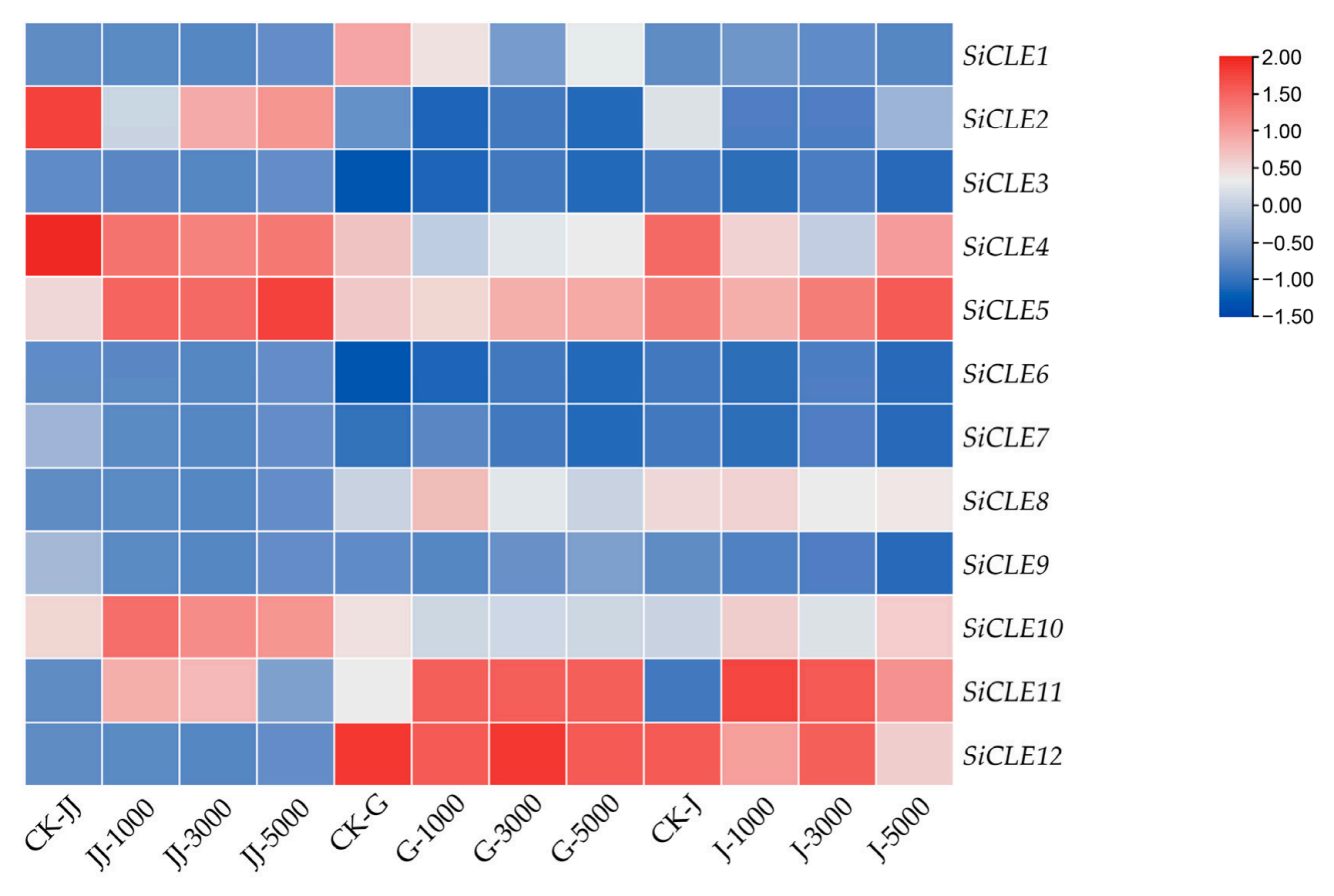
Expression analysis of *CLE* gene family members in response to gravity stress and mechanical trauma in sesame. JJ/J/G-1000/3000/5000 represent the expression changes in shoot tips, stems, and roots under treatments at 1000, 3000, and 5000 rpm. CK-JJ/J/G are the control samples.

**Figure 10:**
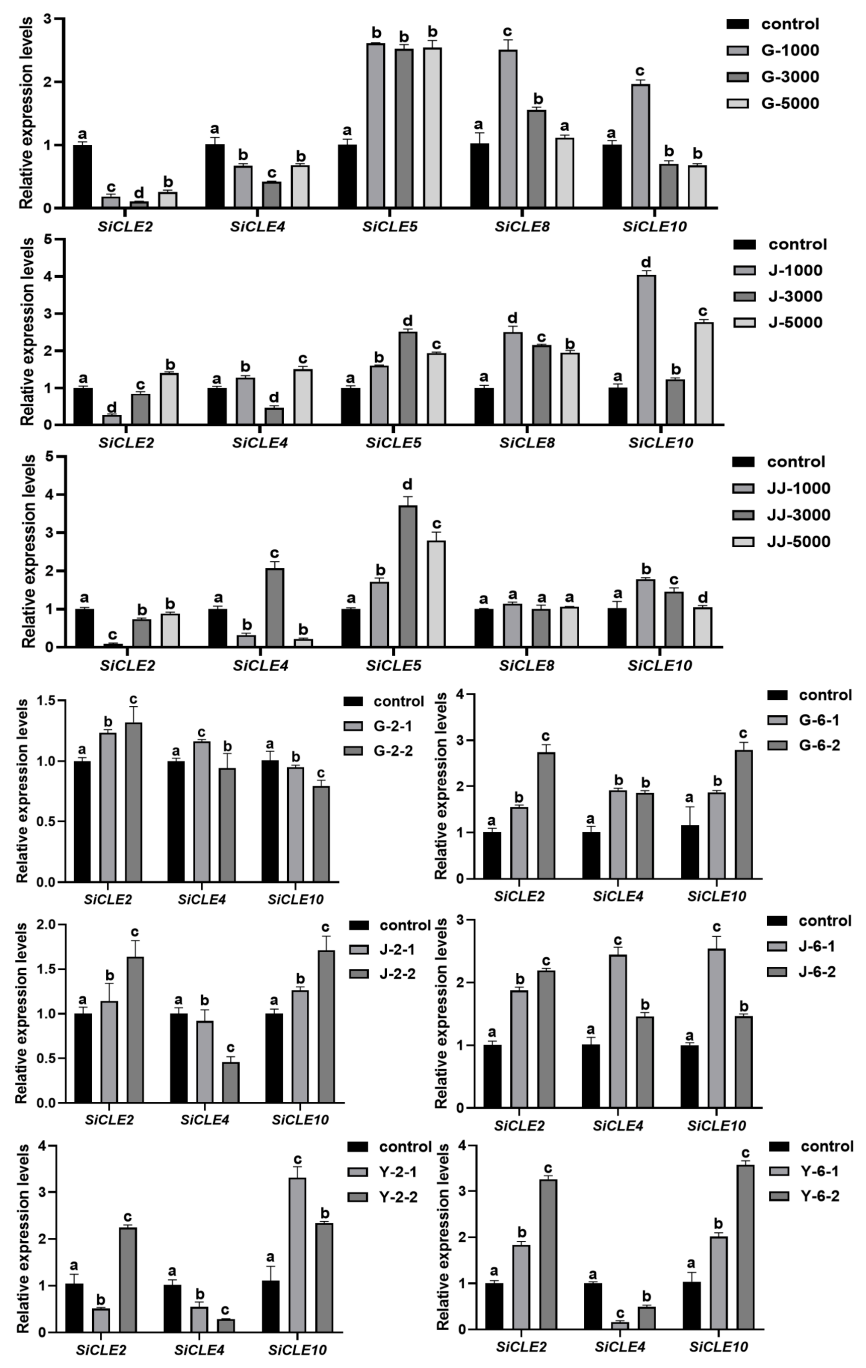
qRT-PCR validation of representative genes. Different lowercase letters indicate significant differences (*p* < 0.05); data are shown as mean ± SEM.

**Table 1:** Information on the physicochemical characteristics, subcellular localization, and conserved motifs of the *CLE* family members in sesame.

Sequence ID	Gene_Name	Length/aa	Molecular Weight	Isoelectric Point	Instability Index	GRAVY	Subcellular Localization	*CLE*_Motif
SIN_1005349.t	*SiCLE1*	82	8838.11	8.55	54.65	0.061	extr	DPDRVAPQGPDPQHH
SIN_1007964.t	*SiCLE2*	133	15,003.39	8.88	47.53	−0.103	extr	VSKRRVPNGPDPIHN
SIN_1016784.t	*SiCLE3*	88	9648.24	6.9	53.37	0.194	chlo	SLFREVPGGPDPRHH
SIN_1018150.t	*SiCLE4*	106	11,510.14	10.95	50.84	−0.598	chlo	STKRKVPNGPDPIHN
SIN_1018188.t	*SiCLE5*	94	10,829.56	10.91	64.77	−0.624	cyto	SSKRKVRRGSDPIHN
SIN_1018226.t	*SiCLE6*	107	11,984.91	11.2	86.27	−0.321	chlo	VEKRFVPSGPNPLHN
SIN_1022558.t	*SiCLE7*	92	10,155.91	10.81	63.93	−0.039	chlo	GTMRVAPGGPDPQHH
SIN_1022559.t	*SiCLE8*	78	8897.14	7.88	81.01	−0.378	extr	DPNRVSPGGPDPKHH
SIN_1022651.t	*SiCLE9*	116	12,592.37	9.18	74.02	−0.549	vacu	VEKRLVPSGPNPLHN
SIN_1026751.t	*SiCLE10*	122	13,243.32	10.86	49.85	−0.111	chlo	MSKRRVPNGPDPIHN
SIN_1027089.t	*SiCLE11*	72	8290.66	10.01	36.27	−0.388	chlo	WPDRVSPGGPDPHHH
SIN_1027090.t	*SiCLE12*	88	9350.84	9.87	38.95	−0.075	vacu	GTKREAPEGPDPQHH

**Table 2:** Prediction of cis-acting elements on the promoters of *SiCLE* genes.

Cis-Element Type	Cis-Element	Sequence	Function
Light-responsive	MRE	AACCTAA	MYB binding site involved in light responsiveness
	chs-CMA1a	TTACTTAA	part of a light-responsive element
	Sp1	GGGCGG	light-responsive element
	3-AF1 binding site	TAAGAGAGGAA	light-responsive element
	GA-motif	ATAGATAA	part of a light-responsive element
	Box 4	ATTAAT	part of a conserved DNA module involved in light responsiveness
	G-Box	(C/T)ACGT(C/G/T)	cis-acting regulatory element involved in light responsiveness
	Gap-box	CAAATGAA(A/G)A	part of a light-responsive element
	I-box	GATAAGGGT	part of a light-responsive element
	AE-box	AGAAAC(AA/TT)	part of a module for light response
	GT1-motif	GGTTAA(T)	light-responsive element
	TCT-motif	TCTTAC	part of a light-responsive element
	ATCT-motif	AATCTAATCC	part of a conserved DNA module involved in light responsiveness
	GATA-motif	AAGGATAAGG	part of a light-responsive element
Abiotic and biotic stresses	ARE	AAACCA	cis-acting regulatory element essential for anaerobic induction
	TC-rich repeats	ATTCTCTAAC/GTTTTCTTAC	cis-acting element involved in defense and stress responsiveness
	GC-motif	CCCCCG	enhancer-like element involved in anoxic-specific inducibility
	MBS	CAACTG	MYB binding site involved in drought-inducibility
	LTR	CCGAAA	cis-acting element involved in low-temperature responsiveness
	CCAAT-box	CAACGG	MYBHv1 binding site
Growth and development elements	O2-site	GATGA(C/T)(A/G)TG(A/G)	cis-acting regulatory element involved in zein metabolism regulation
	GCN4_motif	TGAGTCA	endosperm expression cis-element
	circadian	CAAAGATATC	cis-acting regulatory element involved in circadian control
	AT-rich element	ATAGAAATCAA	binding site of AT-rich DNA-binding protein (ATBP-1)
	CAT-box	GCCACT	meristem expression cis-element
Phytohormone-responsive	ABRE	CGTACGTGCA/TACGGTC/(C)ACGTG	cis-acting element involved in abscisic acid responsiveness
	TGACG-motif	TGACG	cis-acting regulatory element involved in MeJA responsiveness
	TCA-element	TCAGAAGAGG/CCATCTTTTT	cis-acting element involved in salicylic acid responsiveness
	CGTCA-motif	CGTCA	cis-acting regulatory element involved in MeJA responsiveness
	AuxRR-core	GGTCCAT	cis-acting regulatory element involved in auxin responsiveness
	TGA-element	AACGAC	auxin-responsive element
	TGA-box	TGACGTAA	part of an auxin-responsive element
	P-box	CAACAAACCCCTT	gibberellin-responsive element
	TATC-box	TATCCCA	cis-acting element involved in gibberellin responsiveness
	GARE-motif	TCTGTTG	gibberellin-responsive element

**Table 3:** Primer sequences for RT-qPCR.

Gene ID	Gene Name	FP	RP	Product
reference gene	Histone	CTTGATCAGGAAGTTGCCTTTTC	CCTGAAGCGCCAACACAGCAT	101 bp
SIN_1007964.t	*SiCLE2*	TTCCGTCAACCGCAAGTGTT	TGGCAATCTTGAACTCCCCAC	226 bp
SIN_1018150.t	*SiCLE4*	TGGTGGGAGTGTTTCGGTTC	CGGTGGTTGACGAGAGCTAC	203 bp
SIN_1018188.t	*SiCLE5*	TTGTGTAGGGTTACTGGCACTG	CTGGTGCTGGTTTCTTGTCTGT	167 bp
SIN_1022559.t	*SiCLE8*	GTCACATGCCCACATGGCT	CTGCGGTTTCTCTTCTCCTCAG	176 bp
SIN_1026751.t	*SiCLE10*	ACGGAAAACGAAGGACGGTG	GGAGATTGAAAACCACAGACACAC	219 bp

## Data Availability

The transcriptomic dataset analyzed in this study is publicly available from the NCBI Gene Expression Omnibus (GEO) under accession number GSE133186.
